# Health care financing and the sustainability of health systems

**DOI:** 10.1186/s12939-015-0208-5

**Published:** 2015-09-15

**Authors:** Lycourgos Liaropoulos, Ilias Goranitis

**Affiliations:** Center for Health Services Management and Evaluation, Faculty of Nursing, University of Athens, 123 Papadiamantopoulou Street, 11527 Athens, Greece; Health Economics Unit, Public Health Building, University of Birmingham, Birmingham, B15 2TT UK

## Abstract

The economic crisis brought an unprecedented attention to the issue of health system sustainability in the developed world. The discussion, however, has been mainly limited to “traditional” issues of cost-effectiveness, quality of care, and, lately, patient involvement. Not enough attention has yet been paid to the issue of who pays and, more importantly, to the sustainability of financing. This fundamental concept in the economics of health policy needs to be reconsidered carefully. In a globalized economy, as the share of labor decreases relative to that of capital, wage income is increasingly insufficient to cover the rising cost of care. At the same time, as the cost of Social Health Insurance through employment contributions rises with medical costs, it imperils the competitiveness of the economy. These reasons explain why spreading health care cost to all factors of production through comprehensive National Health Insurance financed by progressive taxation of income from all sources, instead of employer-employee contributions, protects health system objectives, especially during economic recessions, and ensures health system sustainability.

## Introduction

Health systems appeared after 1950, as Europe was healing from the 2^nd^ World War. With a political shift to the left [1], governments responded to public demands for affordable health services accessible to all. Until the 1970’s, health systems shared one concern: how to funnel an average 7 % of national Gross Domestic Product (GDP) collected through taxes and labor contributions into health care services. Two major types of public health systems emerged, named after their political instigators:

- *Bismarck systems* based on social insurance, with a multitude of public insurance funds, financed by employer-employee contributions, independent of health care provision. Examples are Belgium, France and Germany.
- *Beveridge systems*, where public financing and health care delivery are handled within one tax-financed structure, such as the National Health Service (NHS) in the UK and in some Nordic states.

Since then, there has been intense debate over the two generic types of systems, with the discussion centered on access, quality and cost. Financing was a *“function of a health system concerned with the mobilization, accumulation and allocation of money to cover the health needs of the people, individually and collectively”* [2]. In the 2000 report of the World Health Organization (WHO) we find that the purpose of health financing was *“to make funding available, as well as to set the right financial incentives to providers to ensure that all individuals have access to effective public health and personal health care”* [2]. The definition was expanded in 2007 as follows: *“A good health financing system raises adequate funds for health, so that people can use needed services protected from financial catastrophe or impoverishment associated with having to pay for them. It provides incentives for providers and users to be efficient”* [3]*.*

In both WHO definitions, the main concern was about raising adequate funds, sidestepping the implications for payers and for the economy. With recent recessions, however, universal coverage, a main pillar of social cohesion and welfare is endangered, with profound implications on equity^1^ and financial protection. The willingness of society to disburse the necessary funds in developing countries has been discussed since the 1980s [4], and sustainable development remains pertinent in light of social, demographic and epidemiological changes [5]. In the developed world, however, the ability to finance society's health care needs is a “child” of the 21^st^ century. The incidence of financing and health system viability has only recently become a major topic of health policy [6], not only in Europe [7] and the UK [8] but also in the US [9] and Canada [10].

The Organization for Economic Co-operation and Development (OECD) opened the debate on financial sustainability in 2013^2^, along with other initiatives at a European Union (EU) level referring to “sustainable health care”^3^. Non-profit organizations, patient advocates as well as the pharmaceutical industry organize workshops and conferences on “access to care” and “patient empowerment” [11]. The 2014 OECD Meeting, held on 24–25 April in Paris, aimed to identify and disseminate good practices in managing health care budgets^4^, and a publication on the fiscal sustainability of health systems is under development. This shall examine drivers of health expenditure, policies to manage spending and improve value for money. Although these are mostly supply-side concerns, the request from the 2013 OECD Meeting was that the 2014 Meeting must also focus on “the politics of reform in health care”, including the issue of demand.

It is difficult to think of a more “political” issue than the source of financing health care. This fundamental, but rather overlooked, concept in the economics of health policy needs to be actively debated as sustainable development goals gain traction in post-2015 policy agenda. This paper discusses the implications of the way health care resources are raised, pooled and spent. Financial sustainability as a major health care issue in the 21^st^ century world is also discussed.

### The debate on sustainability: new challenges in the 21^st^ century

The evolution of health financing during the last half century reveals a fundamental shift in core issues. After 1950, health systems were designed for populations expected to live for an average of 65–70 years. With retirement at 60–65 and near full employment, lifetime earnings and savings were more or less sufficient to finance a decent health system, while rising health expenditure meant welfare gains for all. In the 21^st^ century, average life expectancy rose above the age of 80, and health science and technology improved quality of life even at a very old age. Although desirable, the prolongation of life in good health costs, a reality that no democratic society can ignore for long.

The real political, economic and ethical question is the source of the required financing. Very rich countries^5^ can still afford to rely largely on private health insurance despite the serious equity issues involved. Most developed and developing countries, however, finance their more or less developed welfare state through taxation and labor contributions. It is in these countries that globalization is bringing increasing economic inequality and economic uncertainty has caused a major debate on the sustainability of health financing.

#### Globalization and income inequality

Globalization has profoundly affected the distribution of income both among and within countries. The seminal work of Thomas Piketty in 2014 [12] showed that globalization favors capital relative to other sources of income, such as labor and rents. Increased capital mobility pulled many countries out of poverty, but the benefits favor the rich capital owning countries [13]. Globalization also increased income inequality within countries with top income brackets absorbing a larger share of national GDP [14]. Besides being a moral and political question, growing inequality is also an economic one since, beyond a certain point, it can be a source of significant economic ills [15]. For example, the failure to tax income reduces the effectiveness of welfare and safety nets and undermines the competitiveness of the economy [16]. This point is particularly important for developing countries now developing their health systems.

#### Recession and economic uncertainty

Another phenomenon that makes this century different is frequent recessions as income inequality causes a drop in demand [15]. Unemployment and economic distress put a strain on public budgets, increase the demand for public health services, and limit access to private services [17]. Such extreme pressures, as after the 2008-economic crisis, introduced financial sustainability in the health policy debate. Although the debate is still centered on funding and value for money, it now includes the ability of a society to fulfill its implicit or explicit promise to satisfy need-based demand for health care [18].

### Financing sustainable health care: who must pay and how?

The answer to the question of who must pay for health care and how lies in the moral fabric and the value system of a society. It is a deeply ideological and political question with undertones of social involvement, personal responsibility, and freedom of choice. Big changes in health care financing happen rarely, usually after major events^6^, and are more likely to take place in countries with social cohesion high on their value scale^7^. This is possibly why discussions on health system sustainability continue to “finesse” the question of financing, and perhaps to avoid two uncomfortable truths. One, that reliance on out-of-pocket expenditure is not acceptable on equity and financial protection grounds. Two, that only some kind of income transfer, such as taxation, can cover the increasing cost of health care.

The moral determinant of “who pays” and “how” must now gain importance, as ageing societies, technological advances, globalization, and economic recessions put a strain on the sustainability of financing sources. The question therefore should now focus, not only on whether society as a whole will bear the cost but also on how to obtain and manage the needed savings, and on the efficiency and competitiveness of the economy which must produce them.

For the increasing cost of care many “blame” the demographic factor, although the major part of life-time health cost occurs in the last two years of life [19]. Life expectancy indeed rose significantly in the last fifty years together with total lifetime cost [20]. The average retirement age, however, remained more or less the same at around 65. There are, therefore, twenty years in which a citizen incurs health costs without producing income as “insurance”. People of working age today must finance the health needs of their children, themselves and, mainly, the 3^rd^ and 4^th^ generation. Labor contributions legislated thirty years ago are clearly not enough for today’s medical costs^8^, while contributions sufficient to cover health costs thirty years from now would make labor extremely expensive. Therefore, only savings in the form of taxes on all incomes produced by society, including wealth and capital, appear to be a sustainable source of funding in the long-term.

In addition, cyclical fluctuations are now common events rather than rare occurrences. Health financing may determine how pressures on health systems are weathered without loss of equity, quality and financial protection. Social Health Insurance has been found to have negative labor market effects [21] and to hurt competitiveness [7] due to higher labor costs. This is crucial in monetary unions where devaluation during economic crises is not an option and competitiveness gains are the only way for the economy to adjust to pre-crisis levels. In addition, as unemployment increases, incomes decline and pressures on health budget and public infrastructure are pushed to extremes, evidence has indicated that public health systems financed through taxation can be more responsive to economic pressures and more effective in health expenditure consolidation [22]. Although conclusive evidence is lacking, the experiences of Canada and Greece may be indicative.

Evidence from Canada, where health is financed mainly through taxation, suggests that patient satisfaction, hospital performance and health outcomes were maintained despite the financial strain [23]. Concerns that reliance on taxation may be associated with higher private payments, especially during economic downturns [22], or that corruption may inhibit administrative capacity to collect taxes [24], may be put to rest by the fact that during economic turmoil individuals become more price-sensitive and administrative capacity tends to improve.

In Greece, Social Insurance historically covered approximately 40 % of health care cost. In the face of severe unemployment (27 %) caused by 25 % GDP contraction, reliance on employer-employee contributions proved an inadequate funding base for health care. Between 2009 and 2012,^9^ Social Insurance expenditure declined by 29.3 %, with the fairness of the system and quality of care severely affected [25, 26]. Greece is now a country where the need of re-orientation of health care financing is pressing [25, 27].

In conclusion, employment contributions as a source of health financing are incompatible with universal coverage, quality of services, and rising life expectancy. A move towards general taxation to meet health care needs can boost economic growth through increased competitiveness, and achieve major non-health objectives, like equity, financial protection, quality and responsiveness even during economic downturns. Health system sustainability, as a system objective, must turn to financing through progressive taxation of all types of income. “Uncomfortable” as this may appear, it is a reality not to be overlooked. Political concerns associated with economic imperatives as well as moral considerations may force changes in health services financing in both the developed and developing world. National health insurance financed through taxation should gain momentum in the quest for more sustainable and responsive health systems.

## Footnotes

1 In this paper we treat the concept of Equity-in-Health as implying equal treatment for equal needs, regardless of income or financial ability

2 OECD 2^nd^ Meeting of the joint Network on Fiscal Sustainability of Health Systems, Paris, 25–26 March 2013

3 A conference was organized in Brussels on May 26–27, 2013, as a multi-stakeholder public debate entitled *“From Crisis to Recovery: how to drive sustainable healthcare together?”*. The full Report will be submitted to the European Parliament in early 2015.

4 OECD 3^rd^ Meeting of the Joint DELSA/GOV Network on Fiscal Sustainability of Health Systems, Paris, 24–25 April 2014.

5 Such as the US and Switzerland.

6 For example, the British government in 1947 assumed the full burden of the National Health Service as *“a reward to the British people after the pain and devastation of the war”* [1].

7 The importance of the moral determinant is clear in a comparison of post-war UK, with the US, a country with similar cultural background, fifty years later. The Affordable Care Act – (ACA) of President Obama, although presented as a major health reform, is only a mere extension of government financing to meet the health needs of the 15 % of uninsured poor Americans. Even so, it has become the main issue in the ideological and political warfare in the US.

8 Medical progress is desirable, but it is also expensive and not predictable.

9 2012 is the last year for which official data exist.
